# Lucerastat, an iminosugar with potential as substrate reduction therapy for glycolipid storage disorders: safety, tolerability, and pharmacokinetics in healthy subjects

**DOI:** 10.1186/s13023-017-0565-9

**Published:** 2017-01-14

**Authors:** N. Guérard, O. Morand, J. Dingemanse

**Affiliations:** 1 Department of Clinical Pharmacology, Actelion Pharmaceuticals Ltd, Gewerbestrasse 16, 4123 Allschwil, Switzerland; 2 Department of Global Clinical Science & Epidemiology, Actelion Pharmaceuticals Ltd, Gewerbestrasse 16, 4123 Allschwil, Switzerland

**Keywords:** Lucerastat, Safety, Tolerability, Pharmacokinetics

## Abstract

**Background:**

Lucerastat, an inhibitor of glucosylceramide synthase, has the potential to restore the balance between synthesis and degradation of glycosphingolipids in glycolipid storage disorders such as Gaucher disease and Fabry disease. The safety, tolerability, and pharmacokinetics of oral lucerastat were evaluated in two separate randomized, double-blind, placebo-controlled, single- and multiple-ascending dose studies (SAD and MAD, respectively) in healthy male subjects.

**Methods:**

In the SAD study, 31 subjects received placebo or a single oral dose of 100, 300, 500, or 1000 mg lucerastat. Eight additional subjects received two doses of 1000 mg lucerastat or placebo separated by 12 h. In the MAD study, 37 subjects received placebo or 200, 500, or 1000 mg b.i.d. lucerastat for 7 consecutive days. Six subjects in the 500 mg cohort received lucerastat in both absence and presence of food.

**Results:**

In the SAD study, 15 adverse events (AEs) were reported in ten subjects. Eighteen AEs were reported in 15 subjects in the MAD study, in which the 500 mg dose cohort was repeated because of elevated alanine aminotransferase (ALT) values in 4 subjects, not observed in other dose cohorts. No severe or serious AE was observed. No clinically relevant abnormalities regarding vital signs and 12–lead electrocardiograms were observed. Lucerastat C_max_ values were comparable between studies, with geometric mean C_max_ 10.5 (95% CI: 7.5, 14.7) and 11.1 (95% CI: 8.7, 14.2) μg/mL in the SAD and MAD study, respectively, after 1000 mg lucerastat b.i.d. t_max_ (0.5 – 4 h) and t_1/2_ (3.6 – 8.1 h) were also within the same range across dose groups in both studies. Using the Gough power model, dose proportionality was confirmed in the SAD study for C_max_ and AUC_0–∞_, and for AUC_0–12_ in the MAD study. Fed-to-fasted geometric mean ratio for AUC_0–12_ was 0.93 (90% CI: 0.80, 1.07) and t_max_ was the same with or without food, indicating no food effect.

**Conclusions:**

Incidence of drug-related AEs did not increase with dose. No serious AEs were reported for any subject. Overall, lucerastat was well tolerated. These results warrant further investigation of substrate reduction therapy with lucerastat in patients with glycolipid storage disorders.

SAD study was registered on clinicaltrials.gov under the identifier NCT02944487 on the 24^th^ of October 2016 (retrospectively registered). MAD study was registered on clinicaltrials.gov under the identifier NCT02944474 on the 25^th^ of October 2016 (retrospectively registered).

**Trial registration:**

A Study to Assess the Safety and Tolerability of Lucerastat in Subjects With Fabry Disease. Clinicaltrials.gov: NCT02930655.

## Background

Lysosomal storage disorders (LSDs) are inborn metabolic multisystemic conditions affecting lysosomal functions, characterized by high morbidity and mortality. LSDs comprise more than 50 rare disorders, affecting children, adolescents, and adults, and are caused by specific mutations in genes encoding lysosomal enzymes and transporters. These genes are responsible for the degradation of a wide variety of glycosphingolipids, oligosaccharides, proteins and glycoproteins [1]. Among them, inborn errors of glycosphingolipid (GSL) catabolic enzymes or transporters, lead to diseases collectively termed glycosphingolipidoses or glycolipid storage disorders (GLSDs), and include Gaucher disease, Fabry disease, GM1/GM2 gangliosidoses, Krabbe disease, metachromatic leukodystrophy, and Niemann-Pick Type C disease (NP-C) [2]. In GLSDs, partial or complete deficiency of GSL catabolic enzymes or transporters is associated with the cytotoxic accumulation of specific GSLs, which results in dysfunction of various cell types and damage in multiple organs.

Lucerastat or N-butyldeoxygalactonojirimycin ((2R,3S,4R,5S)-1-butyl-2-(hydroxylmethyl)piperidine-3,4,5-triol) is a soluble, low molecular weight, orally available, iminosugar that has the potential to provide substrate reduction therapy (SRT) for the treatment of GLSDs. The goal of SRT with oral lucerastat is to inhibit the enzyme glucosylceramide synthase (GCS) that catalyzes the first committed step of GSL biosynthesis, thereby reducing the rate of synthesis of downstream GSLs to restore the intracellular balance of synthesis, degradation, and transport of GSLs. The expected net result is a reduction of cytotoxic GSL levels in tissues, thereby preventing, stabilizing, or reversing the progressive deterioration in function of affected organs before irreversible damage occurs. SRT has been established as a safe and efficacious therapeutic modality with miglustat, another iminosugar, for the treatment of Gaucher disease Type 1 (GD1) [3, 4] and NP-C [5], and eliglustat for the treatment of GD1 [6].

Lucerastat inhibits GCS with an inhibitory constant (Ki) of 10.6 μM using ceramide as an acceptor [7], as well as the non-lysosomal glucocerebrosidase (GbA2; EC3.2.1.45) [8]. In addition, lucerastat has no affinity for lysosomal glucocerebrosidase (GbA1; EC3.2.1.45) [9]. Lucerastat appears to be more selective than miglustat as it does not inhibit intestinal sucrase-isomaltase, while it is only a weak inhibitor of intestinal lactase [10]. Despite lucerastat inhibiting the glycosylation of ceramide to glucosylceramide, it did not increase levels of ceramides in cultured cells [11].

Lucerastat is able to cross the blood–brain barrier in line with its pharmacodynamic effect observed in a mouse model of GM2 gangliosidosis, in which lucerastat reduced substrate accumulation (GM2) in the brain, and improved neuromotor performance and increased survival [12, 13]. In Fabry mice, lucerastat significantly reduced globotriaosylceramide (Gb3) storage in the kidney and dorsal root ganglia (data on file).

The safety, tolerability, and pharmacokinetics (PK) of lucerastat were investigated in separate single- and multiple-ascending dose (SAD and MAD) studies conducted in healthy male subjects and testing a wide range of doses. In the MAD study, a group of subjects received lucerastat either in the presence or absence of food.

## Methods

### Subjects

Healthy males aged 18–45 years, with a body weight between 50 and 100 kg, and with a body mass index (BMI) of 18–29 kg/m^2^, were eligible for these studies. Subjects were considered healthy based on medical history, physical examination, vital signs measurements (blood pressure, heart rate, and body temperature), 12–lead electrocardiograms (ECG), clinical laboratory tests (hematology, blood chemistry, and urinalysis), serology (hepatitis B, hepatitis C, and HIV), alcohol breath test, and urine drug test, all performed at screening. Subjects had to use an adequate method of contraception during the study and for 4 months after the follow-up visit. Healthy subjects were not eligible if they had used any prescribed medication within 5 days prior to study entry in the SAD study and within 2 weeks before starting the MAD study. Over-the-counter medications were prohibited within 5 days prior to study start. Other exclusion criteria were: smoking more than ten cigarettes per day, blood loss > 400 mL in the 12 weeks period preceding the study, hypersensitivity to any drug, presence or history of allergy requiring treatment, and existence of any surgical or medical condition which, in the judgment of the clinical investigator, might interfere with the absorption, distribution, metabolism, or excretion of lucerastat. Subjects also had to refrain from strenuous exercise, grapefruit juice and alcohol consumption, from 48 h before admission to the clinical unit until after the follow-up visit.

### Study design

All participants provided written informed consent prior to enrolment in these studies, which were conducted in accordance with the Declaration of Helsinki, Good Clinical Practice, and local regulations. Both studies were approved by the national health authority of United Kingdom and the Edinburgh Independent Ethics Committee for Medical Research. Both studies were single-center, double–blind, randomized, and placebo-controlled trials conducted in healthy males.

Thirty-nine subjects took part in the SAD and were divided in 5 groups: Groups 1 to 4 received a single dose of 100, 300, 500, or 1000 mg of oral lucerastat, respectively, and Group 5 received 2 doses of 1000 mg lucerastat separated by 12 h. In each group, 6 subjects were to receive lucerastat and 2 subjects placebo, except for Group 1 in which only seven subjects were recruited and one placebo dose was not given. All 39 subjects completed the study. After a screening visit performed within 3 weeks prior to dosing, subjects were admitted to the clinic the day before lucerastat administration (Day −1). Subjects received lucerastat in fasted condition in the morning of Day 1. Subjects from Group 1 to 4 were discharged the day after lucerastat intake (Day 2), subjects from Group 5 stayed an additional night at the study site. All subjects returned to the clinical site for a follow-up visit on Day 7. Progression to the next dose group was allowed only after review of all safety data.

In the MAD study, 27 subjects in three different cohorts were planned with, in each cohort, 6 subjects receiving 200, 500, or 1000 mg lucerastat b.i.d. and 3 subjects receiving placebo. In view of some safety findings, the 500 mg dose was repeated to include 10 additional subjects (6 lucerastat and 4 placebo). Thus, Cohort 1 to 4 received 200, 500, 500, and 1000 mg b.i.d. lucerastat, respectively. Subjects were admitted to the clinical unit on Day −1 and received a single dose of lucerastat or placebo in a fasted state b.i.d. from Day 1 to Day 7, i.e., 14 doses in total. Subjects were discharged 36 h after the last lucerastat dose (on Day 9) and returned for a follow-up visit on Day 14. Progression to an increased dose of lucerastat was permitted only after a satisfactory review of all data from the previous cohort. Subjects from Cohort 2 participated in the evaluation of a food effect treatment period before the MAD study per se. These subjects were admitted to the clinical site on the morning of the day before dosing as planned for the MAD study and received a single dose of 500 mg lucerastat in the morning within 15 min of consuming a standardized high-fat breakfast, in line with published guidance on food-drug interactions assessment [14]. Subjects were discharged 24 h after dosing and underwent a washout period of at least 5 days before re-admission to the multiple-dose treatment period. Data from Day 1 of the MAD study were used for the fed vs fasted comparison. Results of the MAD study are presented by dose cohort (200, 500, and 1000 mg) except for the food effect for which Cohort 2 is analyzed independently.

### Clinical supply management

The investigational product (lucerastat) and matching placebo were manufactured applying appropriate Good Manufacturing Practice (GMP) standards, and provided by the sponsor along with a certificate of analysis, including expiry or retest date. Lucerastat and placebo were supplied to the clinical site at least 7 working days before the start of both studies. The sponsor packaged the investigational product and matching placebo to prevent contamination or deterioration during transport and storage. The sponsor determined acceptable storage temperatures, conditions, and times for the investigational product and placebo, and informed the clinical site of these.

### Sample collection & bioanalysis

In the SAD study, for the determination of plasma concentrations of lucerastat, blood samples were collected immediately before dosing (0 h) and at 0.5, 1, 1.5, 2, 2.5, 3, 3.5, 4, 6, 8, 10, 12, and 24 h post-dose in all subjects. In Group 5, additional samples were drawn after the second dose following the same sampling scheme as for the first dose (until the 12 h sample). Lucerastat concentration was also determined in urine samples collected pre-dose and 0–4, 4–8, 8–12, and 12–24 h post-dose. In Group 5, additional urine samples were collected 24–36 and 36–48 h after the first dose.

In the MAD study, for the determination of plasma concentrations of lucerastat, blood samples were collected pre-dose (0 h) and at 0.5, 1, 1.5, 2, 2.5, 3, 3.5, 4, 6, 8, 10, 12, 12.5, 13, 13.5, 14, 14.5, 15, 15.5, 16, 18, 20, 22, and 24 h post-dose on Day 1 and Day 7. Additional blood samples were drawn at 36 h and 48 h after first lucerastat intake on Day 7. Further, trough samples were collected before the morning dose on Day 4 and Day 6. Urine samples were also collected for lucerastat PK 0–6, 6–12, 12–18, and 18–24 h post-dose on Day 1 and Day 7.

Plasma and urine concentrations of lucerastat were determined using a validated liquid chromatography with tandem mass spectrometry (LC–MS/MS) assay with a lower limit of quantification (LOQ) of 50.0 ng/mL in plasma and 1 μg/mL in urine. For plasma samples, inter-run accuracy was between 92.9–102.1% and precision was < 12.3%. For urine samples, inter-run accuracy was between 90.7–103.4% and precision was < 15.1%.

### Safety assessments

In the SAD study, to assess the safety of lucerastat, adverse events (AEs) were recorded from the admission on Day −1 up to the follow-up visit on Day 7. The study investigator assessed the intensity of each AE and its relationship to the study treatment. For all subjects, on Day −1, vital signs, clinical laboratory tests, urine drug screen, and alcohol breath test were performed. Other safety assessments, i.e., 12–lead ECG and vital signs were performed pre-dose as well as at 2, 4, 8, and 24 h post-dose for Groups 1 to 4 and also at 48 h after the first dose in Group 5. Clinical chemistry was repeated at 24 h post-dose for subjects in Groups 1 to 4, and at 48 h post-dose for subjects in Group 5.

In the MAD study, AEs, whose intensity and potential relationship to lucerastat was evaluated by the investigator, were reported from Day 1 up to the follow-up visit. Other safety assessments, including body weight, clinical laboratory tests, vital signs, renal clearance, urine drug test, and alcohol breath test, were performed at admission. Vital signs were assessed pre-dose, at 2, 4, 6, 12, and 24 h post-dose on Day 1 and Day 7, and at 48 h post-dose on Day 7. 12–lead ECGs were recorded (i) pre-dose on Day 1, 3, 5, and 7 (ii) at 2, 4, 12, and 24 h post-dose on Day 1 and 7 (iii) 48 h post-dose on Day 7. Clinical laboratory tests were performed pre-dose at Days 4 and 7, and 48 h after last lucerastat intake on Day 7. On Day 7, body weight was measured pre-dose and physical examination was conducted 48 h post-dose. Because of the well-described gastrointestinal (GI) adverse effects of miglustat [15], stool frequency and consistency were recorded by dose level, subject, and day.

### Pharmacokinetic and pharmacodynamic assessments

In both studies, the plasma PK parameters of lucerastat were derived by non–compartmental analysis (Phoenix WinNonlin - version 3.1) of the concentration–time profiles. All plasma concentration values below the limit of quantification (BLQ, 50.0 ng/mL) were set to zero as there were no BLQ values between two quantifiable values. Reports of BLQ at the end of a concentration-time profile with no samples at subsequent timepoints containing quantifiable concentrations were ignored for PK purposes. Mean concentration–time profiles were generated using these criteria.

The measured individual plasma concentrations of lucerastat were used to directly obtain the maximum observed plasma concentration (C_max_) and the time to C_max_ (t_max_). The area under the plasma concentration–time curve from zero until the last quantifiable concentration (AUC_0-t_) and AUC_0–12_, the area under the plasma concentration–time curve over a dosing interval (12 h), were calculated according to the linear trapezoidal rule, using the measured concentration–time values above the limit of quantification (LOQ). The area under the plasma concentration–time curve from zero to infinity (AUC_0–∞_) was calculated by combining AUC_0-t_ and AUC_extra_. AUC_extra_ represents an extrapolated value obtained by C_t_/λ_z_, where C_t_ is the last plasma concentration above the LOQ and λ_z_ represents the terminal elimination rate constant determined by log–linear regression analysis of the measured plasma concentrations in the terminal elimination phase. The terminal elimination half-life (t_1/2_) of lucerastat was calculated by regression analysis of the terminal elimination slope (t_1/2_ = ln2/λ_z_). Ae was the cumulative amount of unchanged drug excreted in urine and was evaluated in both studies.

In the MAD study, C_max_ and t_max_ were determined after the first dose on Days 1 and 7, and also following dosing on Day 1 of the food effect session.

### Statistical analysis

Summary statistics (i.e., mean, standard deviation [SD], minimum, maximum, number of subjects [n], and coefficient of variation) were calculated for plasma concentrations at each timepoint and each dose level. The same parameters were calculated for urine concentrations in each collection interval and each dose level on each day. Summary statistics were presented for all PK parameters by dose level and day. PK parameters for Cohort 2 were summarized separately by food status. In addition, geometric means and coefficients of variation (based on logarithmically transformed data) were presented for AUC and C_max_ by dose level.

Dose proportionality was assessed across lucerastat doses in the fasted state using the power model described by Gough [16], which was applied to the log e AUC (AUC_0–∞_ for the SAD study and AUC_0–12_ for the MAD study) and C_max_ data. A point estimate and 90% confidence interval (CI) were produced for the population mean slope. Approximate dose proportionality was to be concluded when the 90% CI for the slope was completely within the equivalence boundaries of (0.90, 1.10) for the SAD study and (0.86, 1.14) for the MAD study. Equivalence boundaries were calculated according to the following equation: [1 + ln(ɵ_L_)/ln(dose ratio), 1 + ln(ɵ_U_)/ln(dose ratio)], using the default boundaries of bioequivalence (ɵ_L_ = 0.8, ɵ_U_ = 1.25) as an interval [17].

Additionally, for Cohort 2 in the MAD study, Day 1 C_max_ and AUC_0–12_ values were subjected to ANOVA, including terms for subject and food regimen, to determine the effects of food. The differences between fed and fasted regimen for C_max_ and AUC_0-t_ were explored using ratios of geometric means and their 90% CI with fasting regimen considered as reference. A linear mixed–effects model with the group of subjects as a fixed effect was used for the generation of ratios of geometric means and their 90% CI. Food effect on treatment relative to a fasting regimen was concluded when the 90% CI of the ratio of fed to fasted was outside the 0.8–1.25 range.

## Results

### SAD study

In total, 39 subjects were enrolled in the study. All subjects recruited completed the study. The mean (± SD) age was 29.8 (±7.6) years, the mean weight was 76.4 (±9.8) kg, and the mean height was 178.1 (±7.0) cm. There was no notable difference in mean age, weight, or height between the subjects randomized to placebo and those randomized to lucerastat. Within the 2 weeks before screening, 2 subjects were taking multivitamins and calcium dietary supplements for general health, one subject was treated with erythromycin for acne, and one with paracetamol for headaches. The investigator considered none of these to interfere with the outcome of the study. During the course of the study, one subject randomized to 100 mg lucerastat was administered an analgesic to treat flu symptoms.

During the study, a total of 15 AEs, all of mild intensity, were reported in 10 subjects (Table 1). 2 subjects who received 1000 mg lucerastat b.i.d. developed a total of 5 drug-related AEs. 5 other drug-related AEs were observed in subjects who received placebo (1 AE), 100 mg (2 AEs), or 300 mg lucerastat (2 AEs). All were considered to be “possibly” related to study treatment. The 5 remaining AEs were judged to be unrelated to study treatment. No SAEs were reported for any subject.

**Table 1:** Summary of treatment-emergent adverse events by frequency and preferred term

Single ascending dose study							
	Lucerastat 100 mg (*n* = 6)	Lucerastat 300 mg (*n* = 6)	Lucerastat 500 mg (*n* = 6)	Lucerastat 1000 mg (*n* = 6)	Lucerastat 2 x 1000 mg (*n* = 6)	Placebo Overall (*n* = 9)	Lucerastat Overall (*n* = 24)
Number of subjects with at least one AE	2	2	0	1	4	1	9
Total number of AEs	2	2	0	1	9	1	14
Preferred term							
Fatigue	-	-	-	-	1	-	1
Influenza like illness	1	-	-	-	-	-	1
Constipation	-	-	-	1	-	-	1
Abdominal upper pain	-	-	-	-	1	-	1
Back pain	-	-	-	-	1	-	1
Neck stiffness	-	-	-	-	1	-	1
Cough	-	-	-	-	2	-	2
Hot flushes	-	-	-	-	-	1	-
Headache	-	-	-	-	2	-	2
Paresthesia	1	-	-	-	-	-	1
Somnolence	-	-	-	-	1	-	1
Rash NOS	-	2	-	-	-	-	2
Multiple ascending dose study							
	Lucerastat 200 mg (*n* = 6)	Lucerastat 500 mg (*n* = 12)	Lucerastat 1000 mg (*n* = 6)	Placebo Overall (*n* = 13)	Lucerastat Overall (*n* = 24)		
Number of subjects with at least one AE	3	6	1	5	10		
Total number of AEs	3	7	3	5	13		
Preferred term							
Lymphadenitis submandibular	-	1	-	-	1		
Aphthous stomatitis	-	-	1	-	1		
Constipation	1	-	-	-	1		
Diarrhea	-	1	-	-	1		
Dyspepsia	1	-	-	2	1		
Toothache	1	-	-	-	1		
Nasopharyngitis	-	1	-	-	1		
ALT increased	-	3	-	1	3		
AST increased	-	1	-	-	1		
Dizziness	-	-	-	1	-		
Headache	-	-	1	-	1		
Rash NOS	-	-	1	1	1		

*AE* Adverse Events, Coding details according to MedDRA version 14.0; *NOS* Not otherwise specified

The proportion of subjects with AEs and the number of AEs was highest after dosing with 1000 mg lucerastat b.i.d.. However, 4 of the 5 AEs considered by the investigator to be possibly related to lucerastat were reported after the first dose was given and before the second dose was administered. There was no correlation between the time of onset of AEs and t_max_. AEs observed in the SAD study are summarized in Table 1.

No clinically significant changes from baseline were observed in mean hematology, clinical chemistry or urinalysis values. A number of individual values were above or below the reference range but none were considered clinically significant. All post–dose urinalyses were reported as normal. There was no notable mean change from baseline in any vital sign or ECG parameter assessed and no individual subject value was considered clinically significant. All ECGs recorded during the study were reported as “Normal” or “Abnormal but not clinically significant”. A change from baseline in physical examination was noted for one subject who reported a fine macular rash in the 300 mg group. This AE resolved without sequelae in the absence of treatment.

**Fig. 1:**
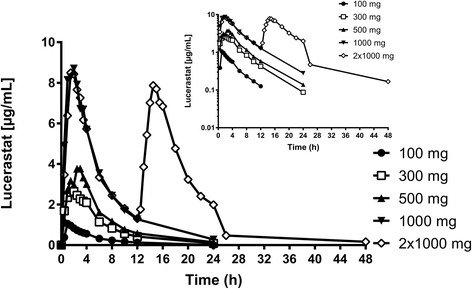
Arithmetic mean plasma concentration–time profiles of lucerastat in healthy subjects after administration of a single oral dose of 100, 300, 500, and 1000 mg lucerastat, or two oral doses of 1000 mg lucerastat separated by 12 h (linear and semilogarithmic scales, 0–48 h). (Per–protocol set)

Administration of increasing doses of lucerastat resulted in a dose-dependent increase in lucerastat plasma concentration (Fig. 1). Peak plasma concentrations of lucerastat were attained between 1.00 and 4.00 h after dosing. In Group 5, in which subjects received two doses of lucerastat 12 h apart (Table 2), 2 subjects reached t_max_ 2.00 and 2.50 h after the second dose. t_max_ exhibited no consistent trend with increasing dose, with median values of 1.25, 2.00, 2.75, 2.25, and 2.00 h for Group 1 to 5, respectively. The range of geometric means of t_1/2_ of lucerastat was 4.43 to 6.47 h among all groups. Lucerastat showed no evidence of accumulation in Group 5 as the second dose of 1000 mg did not result in a higher C_max_ than the first dose given 12 h earlier (Table 2).

**Table 2:** Plasma pharmacokinetic variables of lucerastat

Study	Dose regimen		C_max_ [μg/mL]	t_max_ [h]	AUC_0–12_ [μg · h/mL]	AUC_0–∞_ [μg · h/mL]	t_1/2_ [h]
SAD	100 mg		1.07 [0.83, 1.39]	1.25 [1.00, 3.00]	4.85 [4.13, 5.69]	5.67 [4.77, 6.71]	4.43 [3.59, 5.46]
	300 mg		2.95 [2.17, 4.01]	2.00 [1.50, 4.00]	19.12 [16.27, 22.46]	19.75 [16.81, 23.20]	4.98 [4.65, 5.33]
	500 mg		4.27 [3.75, 4.85]	2.75 [1.50, 3.50]	26.11 [22.94, 29.72]	27.27 [24.13, 30.83]	5.70 [5.11, 6.36]
	1000 mg		10.31 [6.62, 16.05]	2.25 [1.00, 3.50]	57.81 [45.07, 74.14]	60.09 [47.26, 76.41]	5.40 [4.70, 6.22]
	2x1000 mg		10.52 [7.50, 14.74]	2.00 [1.50, 2.50]	116.374 [94.74, 142.96]^1^	118.01 [95.96, 145.12]	6.47 [5.42, 7.72]
MAD	200 mg	Day 1	1.99 [1.61, 2.47]	2.00 [1.00, 3.00]	9.18 [7.35, 11.48]	22.77 [19.26, 26.92]	6.10 [4.78, 7.78]
		Day 7	2.33 [1.68, 3.24]	2.00 [1.50, 3.50]	11.59 [9.72, 13.83]	28.07 [24.69, 31.91]	5.88 [4.68, 7.39]
	500 mg	Day 1	4.82 [4.41, 5.26]	2.50 [1.50, 3.50]	23.20 [20.84, 25.83]	56.87 [50.94, 63.50]	5.84 [4.88, 6.99]
		Day 7	5.18 [4.54, 5.90]	2.50 [0.50, 3.50]	29.37 [25.36, 34.00]	65.83 [56.70, 76.42]	6.33 [5.55, 7.21]
	1000 mg	Day 1	11.39 [9.06, 14.34]	2. 50 [1.50, 3.00]	51.67 [47.47, 56.24]	121.62 [112.03, 132.03]	5.41 [4.05, 7.23]
		Day 7	11.10 [8.69, 14.19]	2.50 [1.50, 4.00]	59.84 [50.97, 70.24]	132.02 [113.28, 153.87]	6.83 [5.76, 8.10]

Data are geometric means (95% CI), except for t_max_, for which medians (range) are given. *C*_*max*_ maximum plasma concentration, *t*_*max*_ time to reach maximum plasma concentration, *AUC*_*0–12*_ area under plasma concentration–time curve over a dosing interval (12 h), ^1^area under plasma concentration–time curve from zero to 24 h; *AUC*_*0–∞*_ area under plasma concentration–time curve from zero to infinity, *t*_*1/2*_ terminal half–life, *CI* confidence interval, *SAD* single ascending dose, *MAD* multiple ascending dose

Data analysis for dose proportionality showed that the 90% CI for the population mean slope was entirely within the acceptance range (0.90, 1.10) for AUC_0–∞_ (0.94, 1.08) and was slightly out of range for C_max_ (0.85, 1.08), suggesting that there was a dose-proportional increase in both variables across the doses tested. Absolute C_max_ and AUC_0–∞_ values are presented in Table 2.

The amount of unchanged drug excreted in urine (Ae) over 12 h after dosing increased in proportion to dose. Mean estimates (SD) were 47 (21), 145 (52), 256 (24), 452 (122), 524 (50) mg, for Group 1 to 5, respectively. Ae data over 12 and 24 h are summarized in Table 3.

**Table 3:** Urine pharmacokinetic variables of lucerastat

Study	Dose regimen		Ae _0-12h_ [21]	Ae _12-24h_ [21]	Ae _0-24h_ [21]	% of dose
SAD	100 mg		47 (21)	6 (4)	53 (24)	53 (24)
	300 mg		145 (52)	10 (6)	153 (54)	49 (20)
	500 mg		256 (24)	24 (12)	280 (12)	56 (6)
	1000 mg		452 (122)	49 (46)	501 (98)	50 (10)
	2x1000 mg		524 (50)	304 (191)	904 (295)	45 (15)
MAD	200 mg	Day 7	160 (14)	145 (31)	305 (40)	76 (10)
	500 mg	Day 7	357 (64)	362 (56)	719 (101)	72 (10)
	1000 mg	Day 7	864 (243)	830 (152)	1695 (289)	85 (15)

Data are arithmetic means (standard deviation); *Ae* cumulative amount of unchanged drug excreted in urine, *SAD* single ascending dose, *MAD* multiple ascending dose

### MAD study

In total, 37 subjects were randomized in the study. All 37 subjects received one or more doses of lucerastat or placebo. One subject was withdrawn from the study in the 500 mg cohort after the morning dose on Day 6 because of increased ALT and AST levels not associated with a bilirubin increase (recorded as moderate and mild severity AEs, respectively, both considered as possibly related to the study drug and above 3 times the upper limit of normal [ULN]). This subject completed the food effect treatment period before beginning the multiple–dose treatment period. All other subjects completed the study. The mean (± SD) age was 27.6 (±6.9) years, the mean weight was 76.1 (±10.4) kg, and the mean height was 179.0 (±6.7) cm. There were no clinically significant differences in mean age, height, weight, or BMI between subjects receiving lucerastat or placebo in any of the dose cohorts. None of the medications taken in the 2 weeks before Screening were considered by the Principal Investigator to influence the outcome of the study, none prevented a subject from entering the study, and all were stopped before the study. During the study, one subject of the 500 mg cohort received 1 g paracetamol to treat cold symptoms.

Eighteen AEs were reported in 15 subjects throughout the study, all but two were considered to be mild in severity. The remaining two AEs were considered to be moderate. One subject in Cohort 2 had nasopharyngitis that was considered to be moderate in severity and unrelated to study drug. This AE began during the food effect treatment and was on-going during the multiple–dose treatment period and resolved without treatment on Day 5. Another subject in Cohort 2 had increased ALT of moderate severity on Day 4 that was considered possibly related to the study drug. Study drug administration was discontinued for this subject after the morning dose on Day 6, when he presented mild severity increase in AST, and was withdrawn from the study on Day 7. In Cohort 2, AEs of increased ALT were recorded in 4 of the 6 subjects (including 1 subject receiving placebo) and all were considered to be possibly related to the study drug. As ALT/AST increased between 2.0–6.1 and 1.2–4.0 fold from baseline in subjects of Cohort 2 for whom AEs of elevated ALT/AST were reported, it was decided to repeat the 500 mg cohort (Cohort 3) before the 1000 mg cohort was dosed. No ALT/AST increase was observed in Cohort 3. Other AEs considered as possibly related to study drug by the investigator were dyspepsia, diarrhea, aphtous stomatitis, dizziness, headache, and skin rash. A slightly higher proportion of subjects with drug-related AEs was reported for subjects dosed with lucerastat compared to placebo-dosed subjects in all dose cohorts (37.5% vs 30.7%) except for Cohort 3 (75.0% vs 16.7%). There was no evidence of an increasing incidence of drug-related AEs with increasing doses of lucerastat. AEs are summarized in Table 1. No SAEs were reported for any subject. No clinically significant changes from baseline were observed regarding vital signs, ECG, hematology, and clinical chemistry (except for AST and ALT elevations mentioned above). An abnormal, clinically significant urinalysis result was reported in a subject who had a trace of blood and of leucocytes in the urine on Day 4. However, this result was not considered an AE by the investigator as the microscopy result was normal, and as the subject had an abnormal, not clinically significant, urinalysis result at admission. A higher proportion of subjects with 2 or more stool samples per study day was observed in Cohorts 2 and 3 compared to subjects receiving placebo or any other dose of lucerastat. However, there was no evidence of an increase in the proportion of subjects with ≥ 2 stool samples over the whole study period.

**Fig. 2:**
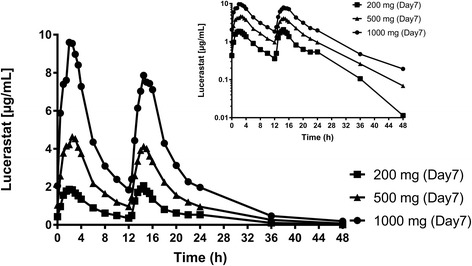
Arithmetic mean plasma concentration–time profiles of lucerastat in healthy subjects on Day 7 after multiple-dose administration of 200, 500, and 1000 mg lucerastat b.i.d. (intakes separated by 12 h, linear and semilogarithmic scales, 0–48 h). (Per–protocol set)

Multiple-dose administration of lucerastat resulted in a dose-dependent increase in lucerastat plasma concentration (Fig. 2).

In the 200 mg cohort, t_max_ occurred between 1.00 and 3.50 h after the second daily dose in 5 out of 6 subjects on Day 1 and Day 7. Median t_max_ was 2.50 h in the 500 mg and 1000 mg b.i.d. dose cohorts, both on Day 1 and Day 7, occurring after the second dose in only one subject. Systemic exposure to lucerastat increased by up to 1.3-fold between Day 1 and Day 7. The time to reach steady state can be inferred from 4 to 5 multiples of t_1/2_ calculated from Day 1 values as approximately 30 h. Thus, steady state was attained on Day 7 of dosing, as confirmed by the pre-dose lucerastat levels on Day 4, 6, and 7. For instance, mean lucerastat plasma trough concentration (± SD) was 2.17 (±0.24), 2.12 (±0.41), and 2.05 (±0.27) μg/mL on Day 4, 6, and 7, respectively.

Analysis of the data from Day 7 for dose proportionality showed that the 90% CI for the population mean slope was entirely within the acceptance range (0.86, 1.14) for AUC_0–12_ (0.91, 1.13) and was slightly out of range for C_max_ (0.82, 1.10), suggesting that there was a dose-proportional increase in both variables across the doses tested. Ae over 24 h after dosing on Day 7 increased in proportion to dose. Mean Ae estimates (SD) were 305 (40), 719 (101), and 1695 (289) mg, for the 200, 500, and 1000 mg dose groups, respectively. Ae data over 12 and 24 h are summarized in Table 3.

GMR of C_max_ and AUC_0–12_ values of Day 1 were calculated to compare the fed and fasted regimens for 500 mg lucerastat (Cohort 2). C_max_ was greater after dosing in the fasted state than after dosing in the fed state, as indicated by a GMR of 0.76 (90% CI: 0.61, 0.94). Total systemic exposure to lucerastat on Day 1 was similar following dosing in the fasted and fed states, with an adjusted GMR of 0.93 (90% CI: 0.80, 1.07). Median values for t_max_ were similar after dosing in the fed and the fasted regimen (2.75 h). Ae was also similar after dosing in both the fasted and fed state, with mean (SD) Ae from 0 to 12 h in fasted state of 433 (148) mg vs 403 (143) mg in the fed state.

## Discussion

Lucerastat was well tolerated in both studies. In the SAD study, there was no difference in the proportion of subjects with drug-related AEs after any treatment, although the number of AEs was highest in the subjects dosed with 1000 mg b.i.d. There was no correlation between the time of onset of AE and lucerastat plasma exposure. Lucerastat was considered to be safe following multiple dosing with 200, 500, and 1000 mg b.i.d. for 7 days and as a single 500 mg dose in the fed state. Increases from baseline in ALT/AST concentrations were observed in 500 mg lucerastat-dosed subjects within Cohort 2 as well as in a placebo-dosed subject. Elevated ALT levels, observed 4 times in Cohort 2, are more specific for liver damage than AST elevations [18], which was reported only one time in the same cohort. Due to the elevations of liver transaminases in the 500 mg b.i.d. Cohort 2, it was decided to repeat this dose level. No notable increases in ALT or AST levels were detected in the subjects in Cohort 3. As the incidence of elevated ALT levels was similar in placebo- and lucerastat-dosed subjects in Cohort 2, it was most likely due to the relative inactivity of the subjects and weight gain during the residential period as described previously. It is known that transaminase increases occur in residential studies also in subjects receiving placebo. In a pooled population from 13 phase I studies, 7.5% of 93 subjects who received placebo presented at least 1 value of ALT that was twice ULN, and 20.4% had at least 1 value above ULN [19]. In the MAD study only 1 subject had an ALT increase > 2 x ULN, i.e., 2.7% of the population of the study, and 10 subjects had at least 1 ALT value above ULN (27% of the subjects enrolled). Therefore, greater attention was paid to the need for regular exercise and prevention of weight gain in Cohort 3 and 4, and the subjects in these cohorts were weighed daily. As there was no ALT/AST increase in these last 2 cohorts and taking into account the role of residential studies in increasing ALT levels in healthy subjects, there was no safety concern regarding elevation of liver transaminases caused by lucerastat in these 2 studies. These data are to be confirmed in studies with larger numbers of patients.

The GI tolerability of lucerastat was good, in contrast to that of miglustat. There was no effect of increasing dose or multiple dosing of lucerastat on stool frequency or on the incidence of GI AEs in healthy subjects. The good GI tolerability of lucerastat has been corroborated in chronic toxicology studies in rodents and dogs (data on file). This differentiation in GI tolerability between the two structurally related compounds can be explained by the fact that lucerastat is a galactose analog iminosugar which does not inhibit intestinal sucrase-isomaltase. Miglustat is a glucose analog iminosugar and a potent inhibitor of sucrase-isomaltase [7, 15].

In the SAD study, the systemic exposure to lucerastat (as indicated by C_max_ and AUC_0–∞_) increased proportionally as the dose was increased from 100 to 1000 mg. This indicates the absence of saturation of absorption or elimination mechanisms, also at high doses. Dose-related increase in systemic exposure was confirmed in the MAD study, for which dose proportionality was concluded statistically regarding AUC_0–12_. All other PK parameters reported were independent of dose. The PK of lucerastat showed relatively low intersubject variability and did not change with time.

The fraction of administered dose excreted as unchanged drug in urine was high, suggesting that renal elimination of lucerastat is the most relevant route of excretion. This had also been seen in previous preclinical ADME studies with radiolabeled lucerastat (data on file) in which excretion of radioactivity via urine was predominant (60% in rats and 67% in dogs), similarly to what was observed with miglustat in rats [20]. C_max_ and AUC_0–12_ values were similar following dosing in the fasted and fed states. Thus, food intake did not affect the total systemic exposure to lucerastat, which may be taken without regard to meals.

In the MAD study, lucerastat plasma concentration was equal to or above the Ki for GCS inhibition for 50% and 80% of the dosing interval at doses of 500 and 1000 mg b.i.d., respectively, which is deemed to be adequate to achieve SRT in patients with GLSDs.

## Conclusion

In conclusion, oral lucerastat was well tolerated in terms of AEs, in particular GI tolerability, and with respect to all safety variables assessed. Based on the favorable safety, tolerability, and PK results of lucerastat in healthy subjects, the potential of lucerastat as SRT was assessed in an exploratory study in subjects with Fabry disease in which safety was evaluated, and plasma and urine levels of several glycosphingolipids were measured (Identifier NCT02930655). The results of this study will be reported separately (21).

## Abbreviations

AE: Adverse event
Ae: Cumulative amount of unchanged drug excreted in urine
ALT: Alanine aminotransferase
AST: Aspartate aminotransferase
AUC: Area under the plasma concentration time curve
AUC_0–12_: Area under plasma concentration-time curve over a dosing interval
AUC_0–∞_: Area under plasma concentration-time curve from zero to infinity
BLQ: Below the limit of quantification
BMI: Body Mass Index
CI: Confidence interval
C_max_: Maximum plasma concentration
ECG: Electrocardiogram
Gb3: Globotriaosylceramide
GbA1: Non-lysosomal glucocerebrosidase
GbA2: Lysosomal glucocerebrosidase
GCS: Glucosylceramide synthase
GD-1: Gaucher disease type 1
GI: Gastrointestinal
GLSD: Glycolipid storage disorders
GMP: Good manufacturing practice
GMR: Geometric mean ratio
GSL: Glycosphingolipid
Ki: Inhibitory constant
LC-MS/MS: Liquid chromatography with tandem mass spectrometry
LOQ: Limit of quantification
LSD: Lysosomal storage disorders
MAD: Multiple ascending dose
NP-C: Niemann-pick type C
PD: Pharmacodynamics
PK: Pharmacokinetics
SAD: Single ascending dose
SAE: Serious adverse event
SD: Standard deviation
SRT: Substrate replacement therapy
t_1/2_: Terminal half-life
t_max_: Time to reach maximum plasma concentration
ULN: Upper limit of normal
